# Identification and Characterization of the *Spodoptera* Su(var) 3-9 Histone H3K9 trimethyltransferase and Its Effect in AcMNPV Infection

**DOI:** 10.1371/journal.pone.0069442

**Published:** 2013-07-24

**Authors:** Binbin Li, Sisi Li, Juan Yin, Jiang Zhong

**Affiliations:** Department of Microbiology and Microbial Engineering, School of Life Sciences, Fudan University, Shanghai, People’s Republic of China; Ludwig-Maximilians-Universität München, Germany

## Abstract

Histone H3-lysine^9^ (H3K9) trimethyltransferase gene *Su*(*var*) *3-9* was cloned and identified in three *Spodoptera* insects, *Spodoptera* *frugiperda* ( *S* *. frugiperda* ), *S* *. exigua* and *S* *. litura* . Sequence analysis showed that *Spodoptera* Su(*var*) *3-9* is highly conserved evolutionarily. Su(var) 3-9 protein was found to be localized in the nucleus in Sf9 cells, and interact with histone H3, and the heterochromatin protein 1a (HP1a) and HP1b. A dose-dependent enzymatic activity was found at both 27 °C and 37 °C in vitro, with higher activity at 27 °C. Addition of specific inhibitor chaetocin resulted in decreased histone methylation level and host chromatin relaxation. In contrast, overexpression of Su(var) 3-9 caused increased histone methylation level and cellular genome compaction. In AcMNV-infected Sf9 cells, the transcription of *Su*(*var*) *3-9* increased at late time of infection, although the mRNA levels of most cellular genes decreased. Pre-treatment of Sf9 cells with chaetocin speeded up viral DNA replication, and increased the transcription level of a variety of virus genes, whereas in Sf9 cells pre-transformed with Su(var) 3-9 expression vector, viral DNA replication slow down slightly. These findings suggest that Su(var) 3-9 might participate in the viral genes expression an genome replication repression during AcMNPV infection. It provided a new insight for the understanding virus–host interaction mechanism.

## Introduction

Post-translational modifications on N-terminus of core histones, such as methylation, acetylation, phosphorylation, ubiquitination and glycosylation, *etc.*, could affect the affinity between histones, DNA and a variety of protein factors, and change the status of chromatin compaction and gene expression [1,2]. Site- and state-specific methylations of histone Lysine or Arginine are catalyzed by a family of proteins containing SET domain, which was first identified in *Drosophila* Su(var) 3-9, Enhancer of zeste and Trithorax [3], from which the acronym is derived. The SET domain is highly conserved among most histone methyltransferases (HMTs). The C-terminal half of SET contains H(x_2_) NHSC and GE(x_5_) Y motifs, which was defined as the catalytic core [4,5]. It is often flanked by one or both of the functionally associated domains named preSET and postSET [3,4]. The preSET, which is also known as SAC (SET-domain-associated cysteine-rich region), interacts with surfaces of core SET to stabilize the structure [3,4], while the postSET contributes to the binding of methyl donor S-adenosylmethionine (SAM) and the methylation [6]. Different histone Lysine methyltransferases (HLMTs) have variable activities to catalyze Lysine methylations at different site (H1K26, H3K4, H3K9, H3K27, H3K36, H3K79 and H4K20), and to different methylation statuses (mono-, di- and tri-methylation).

Su(var) 3-9 is originally defined as *Drosophila* suppressor of variegation and is included in the SUV39 family. It specifically triggers histone H3K9 trimethylation [7–9] and provides binding signal for HP1, thus participates in heterochromatin formation and gene silencing [10–12]. Su(var) 3-9 also interacts with the Chromo-Shadow domain of HP1 through the N-terminus dimerization region [13,14]. Moreover, Su(var) 3-9 interacts with various epigenetic factors such as DNA methyltransferase (DNMT) and histone deacetylase (HDAC) through bridge proteins HP1 and methyl-CpG-binding protein 2 (MeCP2) [15–18], therefore is involved in the complex transcription regulatory network.

Virus infection usually affects multiple biological processes of the host, which may activate host antiviral responses in return. In the perspective of cellular epigenetic regulation, it is known that the genomes of many DNA viruses, including herpesviruses, adenoviruses, hepatitis B virus, are subject to histone binding, and form chromatin-like structure in the host nucleus. On the other hand, virus may make the use of epigenetic mechanisms to ensure their replication [19–23]. The insect baculovirus *Autographa* *californica* multiple nucleopolyhedrovirus (AcMNPV) is known to induces a series of changes in the host cells Sf9, such as cellular cytoskeleton alternation, cell cycle arrest at G2/M, cellular gene transcription inhibition and protein synthesis shut-off, *etc.* [24,25]. It has been suggested that AcMNPV genome also has the nucleosome structure in infected cells [26], and chemical inhibitors of HDAC and DNMT could affect virus genome replication and gene transcription [27–29]. However, the details of AcMNPV-host interaction at the epigenetic level are largely unknown.

In the current study, we identified *Su*(*var*) *3-9* from three *Spodoptera* insects, *S* *. frugiperda* , *S.* exigua and *S* *. litura* . The splice variant of *Su*(*var*) *3-9, eIF2γ*, and two of the three *HP1* variants, *HP1a* and *HP1b* was also identified in *S* *. frugiperda* . The expression and subcellular localization of Su(var) 3-9, and its interaction with Histone H3 substrate, HP1a and HP1b were investigated in Sf9 cells. The enzymatic activity of Su(var) 3-9 was evaluated *in vitro* and *in vivo*. Moreover, the effect of Su(var) 3-9 on virus genes expression and viral genome replication during AcMNPV infection was also studied. Our findings provide a new perspective for the potential roles of a HMT in AcMNPV-insect interaction.

## Materials and Methods

### Cell and larval culture, genome and RNA extraction, and complementary DNA generation

*S* *. frugiperda* cell line Sf9 was cultured at 27 °C in TMN-FH medium supplemented with 10% fetal bovine serum (FBS), 100 U/ml penicillin and 100 µg/ml streptomycin. *S* *. exigua* and *S* *. litura* larva were reared on an artificial diet (Baiyun Industrial Co., Ltd., Henan, China) at 25 °C.

The genomic DNA was extracted using Cellular Genome Extraction Kit (Songon, China) according to the manufacturer’s instructions. Total cellular RNA was isolated using Trizol (Invitrogen, USA) for common applications, or Qiagen RNeasy Mini Kit (Qiagen, USA) for cDNA cloning, followed by DNase I digestion. For larval RNA extraction, fifth instar larvae were frozen in liquid nitrogen followed by repeats of freeze-thawing, and the frozen larvae were grinded. Complementary DNA (cDNA) was generated via reverse transcription with PrimeScript Reverse Transcriptase (Takara Bio, Dalian, China) for cDNA mapping, or PrimeScript RT Master Mix (Takara Bio) for quantification.

### Sequence determination and bioinformatic analysis

The sequence of *B. mori* Su(var) 3-9, HP1a and HP1b (Table S1) were used to search homologues *in* *S* *. frugiperda* against SPODOBASE at the website http://bioweb.ensam.inra.fr/spodobase [30]. Expressed sequence tags (ESTs) that share maximum similarity with that of *B. mori* homologues, were obtained (information listed in Table S1). 5'- and 3'-rapid amplification of cDNA ends (RACE) were performed to clone the full-length cDNA from Sf9 cells using SMARTer RACE cDNA Amplification Kit (Clontech, USA) according to the manufacturer’s instruction, with primers UPM and NUPM from kit, and additional primers listed in Table S2. The coding sequences of Su(var) 3-9/eIF2γ of *S* *. exigua* and *S* *. litura* were amplified with polymerase chain reaction (PCR) using common upstream primer s/eF and downstream primers sR and eR, respectively. Two fragments generated by PCR with primer pairs GeneF1/GeneR1 and GeneF2/GeneR2 were assembled to get the full length nucleotide sequence of Su(var) 3-9/eIF2γ locus within the genome context in Sf9 cells. All primer sequences above are listed in Table S3.

*S* *. frugiperda* Su(var) 3-9 was aligned with selected homologues from Genbank using Clustal W software [31]. Phylogenetic tree was generated by the Neighbor-Joining method using the alignment on MEGA5 software [32] with 1 000 bootstrap replicates. Percent amino acid identities between selected HMTs were calculated on BioEdit software [33] based on the alignment.

### Plasmid construction

Open reading frames (ORFs) of Su(var) 3-9, HP1a and HP1b, and the partial Su(var) 3-9 ORF excluding the overlapping region with eIF2γ (named as Suv) were amplified with PCR using primer pairs CommonF(-ATG)/Su(var) 3-9R, HP1aF(-ATG)/HP1aR, HP1bF(-ATG)/HP1bR, and SuvF/Su(var) 3-9R, respectively. For all fragments, a 5’ BamHI and a 3’ XhoI were added for easy cloning in the following steps (primers used are listed in Table S2). Prokaryotic expression plasmids pET28a-Su(var) 3-9, pET28a-Suv, pET28a-HP1a, pET28a-HP1b and pGEX-4T-1-Su(var) 3-9 were generated by inserting corresponding PCR fragments into the BamHI/XhoI restriction sites of pET-28a (Novagen, USA) in frame with the N-terminal 6×Histidine (His) tag, or pGEX-4T-1 (Amersham Biosciences, England) in frame with the N-terminal glutathione-S-transferase (GST) tag.

The hr5ie1 fragment comprising AcMNPV hr5 sequence and IE1 promoter was amplified with primer pair hr5ie1F/hr5ie1R (Table S2) using p402 (provided by Dr. Jing Ge, Fudan University) as template, followed with BstZ17I/XhoI digestion. phr5ie1 was generated by inserting hr5ie1 into the BstZ17I/SpeI restriction sites of pFastbac1 (Invitrogen). phr5ie1-Su(var) 3-9 was constructed by inserting the 6× his-Su(var) 3-9 coding region, which was excised from pET28a-Su(var) 3-9 with XbaI/XhoI, into the same restriction sites in phr5ie1.

### Prokaryotic expression and antibody production

Recombinant proteins His-Su(var) 3-9, His-HP1a, His-HP1b, His-Suv, GST and GST-Su(var) 3-9 were expressed in *Escherichia coli* strain Rosetta (DE3) (Novagen, USA) by plasmids pET28a-Su(var) 3-9, pET28a-HP1a, pET28a-HP1b, pET28a-Suv, pGEX-4T-1 and pGEX-4T-1-Su(var) 3-9, respectively. 6× His-tagged proteins were purified with His60 Ni Superflow Resin (Clontech). GST and GST-tagged protein were purified with Glutathione HiCap Matrix (Qiagen). Purified proteins were dialyzed against storage buffer (50 mM Tris-HCl, pH 8.5 at 25 °C, 4 mM DTT, 5 mM MgCl_2_, 100mM NaCl, 50% glycerol) and stored at -80 °C. Protein concentration was determined with Bradford Protein Assay Kit (Songon). All experiments were carried out following the manufacturer’s instructions

Anti-Su(var) 3-9, anti-HP1a and anti-HP1b polyclonal antiserums were generated by immunizing Kunming mice with purified proteins of His-Suv, His-HP1a and His-HP1b, respectively, and IgGs were purified with Protein A/G Affinity Resin (GE, USA). Animal maintenance and experiments were conducted according to the European Community guidelines for the care and use of animals, and approved by the Ethic committee for Animal Care and Use of Fudan University.

### Western blot and immunofluorescent localization

Sf9 cells were harvested by centrifuged at 3 000 rpm for 5 min and washed with phosphate-buffered saline (PBS) (pH7.4) twice. Total cellular proteins were resolved by 12% sodium dodecyl sulfate/polyacrylamide gel electrophoresis (SDS-PAGE) followed by electro-transferred onto nitrocellulose membranes (Millipore, USA). Proper antibodies generated as described above (1:1000), or mouse anti-6xHis (1:2000) (Sigma, USA), rabbit anti-H3 (1:2000) (Active Motif, USA), mouse anti-trimethyl histone H3K9 (anti-H3K9me3) (1:1000) (Millipore) were used as primary antibodies. Alkaline phosphatase-conjugated anti-mouse IgG or anti-rabbit IgG (Sigma) were used as secondary antibody at a dilution of 1:5000.

For immunofluorescence assay, Sf9 cells were seeded onto coverslips in 6-well culture plate at a confluency of approximate 40-50% and anchored overnight before being fixed with cold acetone for 10 min and subsequently permeabilized with 0.2% Triton X-100(v/v). Cells were incubated in blocking solution (10% goat serum and 1% BSA in PBS) for 1h, before being incubated with the primary antibody against Su(var) 3-9 (1:100) for 2 h. The cells were then incubated with tetraethyl rhodamine isothiocyanate (TRITC)-conjugated goat anti-mouse IgG (1:200) diluted with blocking solution for another 2 h. Specimens were covered onto slides with a drop of mounting medium (carbonate-buffered saline (pH8.5) with 90% glycerol (v/v)). Triplicate PBS-washing for 5 min were included in the intervals of each step. The Laser confocal microscope (model TCS-SP equipped with an Ar–Kr laser; Leica, Heidelberg, Germany) was used to examine the specimen.

### Plasmid transfection, HMT inhibitor treatment and AcMNPV infection of Sf9 cells

Sf9 cells were seeded on 24-well culture plate at approximate 5×10^4^ cells per well and cultured overnight before use. Cellfectin II Reagent (Invitrogen) was used for transfection experiments with quantified plasmid DNA following manufacturer’s instruction. For HMT inhibitor (HMTi) experiment, appropriate concentration of Su(var) 3-9-specific inhibitor Chaetocin (Enzo Life Science, USA) [34] determined by MTT assay (Wang, et al. [35]) was added to the culture medium. For AcMNPV infection, Sf9 cells were inoculated at specified multiplicity of infection (MOI) by directly adding virus stock into medium and incubated for 4h at 27°C. The virus inoculum was then removed and replaced with fresh medium.

### Nuclear protein extraction

Nuclear extract was prepared using hypotonic buffer solution (20 mM Tris-HCl, pH 7.4, 10 mM NaCl, 3 mM MgCl_2_) and Cell Extraction Buffer (Cat. no. FNN0011, Sigma) supplemented with 1mM phenylmethylsulfonyl fluoride (PMSF) and Protease Inhibitor Cocktail (Sigma) at recommended concentration, following manufacturer’s instruction. A DNase I digestion step was added in order to release proteins from tightly coupled DNA-protein complex in the nuclear fraction. The final extract was quantified with Bradford method, aliquoted and stored at -80°C.

### GST pull-down and co-immunoprecipitation

The recombinant protein GST-Su(var) 3-9 or GST was used as the bait to capture potential binding partners in the nuclear extract of Sf9 cells. GST or GST-Su(var) 3-9 protein was immobilized on Glutathione HiCap Matrix (Qiagen, USA) resin, and the bound resin was incubated with the nuclear extract following manufacturer’s instruction. Nonspecific bound fraction was rinsed out and the bound fraction was eluted from the matrix and subjected to Western blot analysis.

Rabbit anti-H3 antibody (Active Motif) was used to immunoprecipitate proteins in complex with H3 in the nuclear extract of Sf9 cells. Pre-cleaned nuclear extract of Sf9 cells was incubated with rabbit anti-H3 antibody or irrelevant rabbit IgG, before the antibody-antigen complex was captured and precipitated with PureProteome™ Protein G Magnetic Beads (Millipore), following the manufacture’s instruction. Nonspecific fraction was rinsed out with RIPA buffer (50 mM Tris-HCl pH 7.4, 150 mM NaCl, 1 mM EDTA, 1% NP-40, 0.25% Sodium deoxycholate, 1 mM Na _3_VO_4_, 1 mM NaF, 1 µg/ml aprotinin, 1 µg/ml leupeptin, 1 µg/ml pepstatin, 1 mM PMSF), and the specific fraction was subjected to Western blot.

### HMT enzymatic assay in vitro and in Sf9 cells

HMT enzymatic assay *in vitro* was performed as described by Sun et al. [36] with minor modifications. Briefly, a 25 µl reaction system comprising 1 µg Histone H3.2 (NEB, USA), 160 µM SAM and His-Su(var) 3-9 protein in HMT reaction buffer (50mM Tris-HCl, pH 9.0, 5 mM MgCl_2_, 4 mM DTT) was incubated at 27 °C or 37 °C for 60 min. The reaction was stopped by adding equal amount of 2×SDS-PAGE loading buffer, and the total protein was analyzed by Western blot using specific antibody for trimethylated or non-methylated H3. For *in vitro* HMT inhibition assay, 5 µM Chaetocin was supplemented in the reaction mixture.

HMT activity of Su(var) 3-9 in Sf9 cells was examined using nuclear proteins of variously treated or non-treated Sf9 cells with EpiQuik Histone Methyltransferase Activity/Inhibition Assay Kit (H3K9) (Epigentek, USA) following the manufacturer’s instruction.

### Real-time quantitative PCR

Real-time quantitative PCR (qPCR) was used to determine the changes of host or viral transcription and viral DNA replication in AcMNPV infected Sf9. It was carried out on Mx3000p (Stratagene, USA) using SYBR Premix DimerEraser (Takara Bio). Template cDNA and DNA were prepared as described above. The relative level of transcriptional change was determined using 2^-ΔΔCt^ method [37]. For replication assay, the copy number of viral genome was calculated from the Ct values through a standard curve derived from serial dilutions of AcMNPV DNA stock. Primer sequences of qPCR were listed in Table S4.

### DNase I-sensitivity assay

DNase I-sensitivity studies [27] were performed to evaluate alternation of chromatin compaction. Briefly, Sf9 cells were collected at specific time point post treatment, washed with PBS and resuspended in 300 µl supplemented RSB buffer (20mM Tris-HCl, pH 7.4, 10mM KCl, 1.5mM MgCl_2_, 1mM CaCl_2_, 0.5% (v/v) NP-40, 100µg/ml PMSF, 20µg/ml RNase A). The sample was then split into 50µl aliquots and divided into untreated and treated group equally. The treated group was subjected to DNase I digestion at 37° for 5 min with different concentrations of enzyme, and stopped with the stop buffer (50mM Tris-HCl, pH 8.0, 0.1 M NaCl, 1% (v/v) SDS and 100 mM EDTA). Samples were then incubated with proteinase K overnight, and DNA was extracted with phenol/chloroform and precipitated with ethanol. The percentage of DNA resistant to the nuclease was determined with qPCR with primers for Sf9 *β-Actin* and β*-Tubulin* (Table S4).

### Statistical analysis

Representative results from at least two independent experiments were shown. Data from triplicate or more parallel experiments were used to calculate mean value and standard deviation.

## Results

### Sequence determination of *Spodoptera* Su(*var*) 3-9/eIF2γ

**Figure 1:**
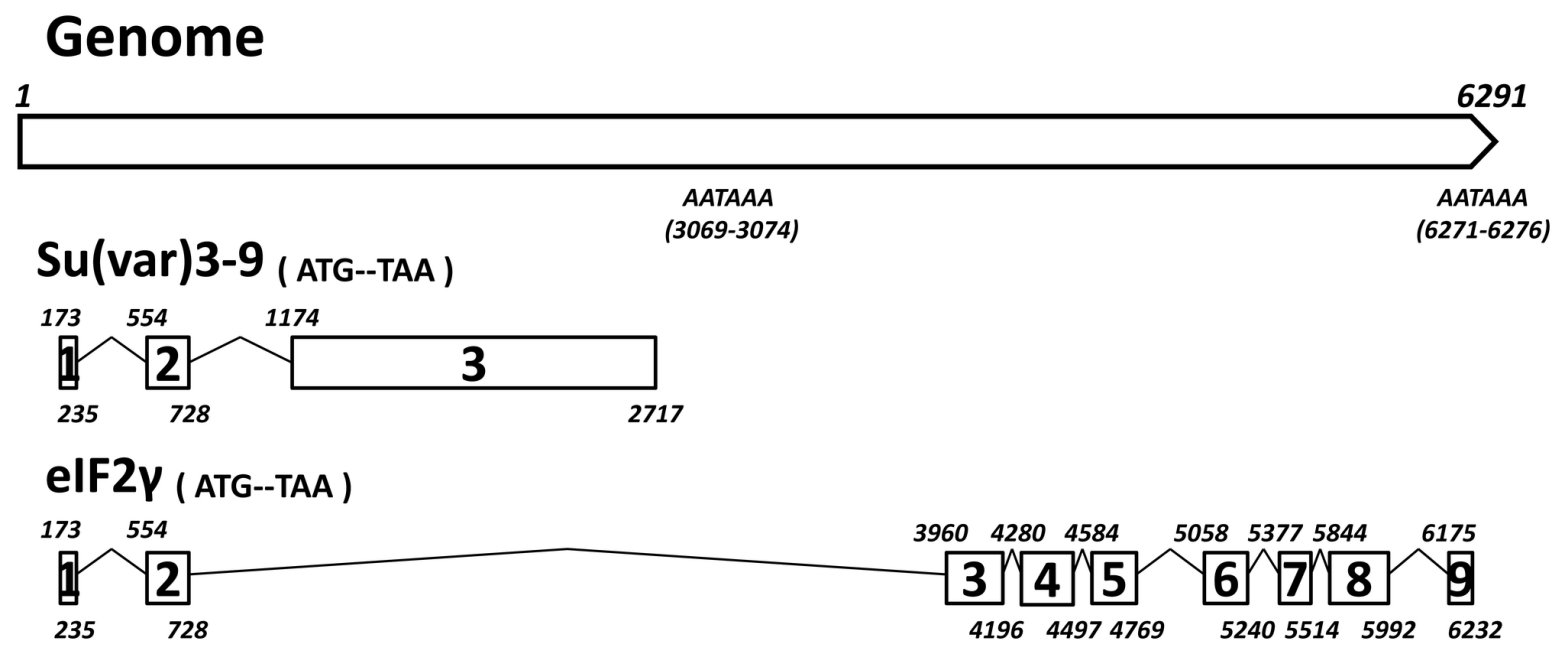
Schematic diagram of *Spodoptera* Su(*var*) *3-9/eIF2γ* genome context and splice pattern. Numbered boxes represent exons of ORFs. The corresponding sites in genome for each exon are indicated (numbers at upper left for starts and lower right for ends). Broken lines between exons represent intron regions. The polyadenylation signals downstream of ORFs are indicated.

In holometabolic insects, *Su*(*var*) *3-9* and its functionally unrelated gene *eIF2γ* are commonly seen as two splice variants [38,39]. The partial EST of *S* *. frugiperda* eIF2γ was found in SPODOBASE [30] using the sequence of *Bombyx mori* homologue. 5’- and 3’-rapid RACE were carried out to obtain the full length sequence of *eIF2γ* transcript. Based on the obtained sequence, a 6 921 bp fragment was amplified from the genome, in which the full coding sequence of *Su*(*var*) *3-9* was identified. It was then verified by PCR with cDNA as template. The sequences of *Su*(*var*) *3-9* and *eIF2γ* were also identified in another two *Spodoptera* insects, *S* *. exigua* and *S* *. litura* . They were 100% identitical to those of *S* *. frugiperda* . The genomic structure of the *Su*(*var*) *3-9*/*eIF2γ* locus and the splicing patterns was shown in Figure 1. There are three exons in *Su*(*var*) *3-9* ORF (1 782 bp, encoding a 593 aa protein) and nine exons in *eIF2γ* ORF (1 665 bp, encoding a 554 aa protein), with the first two exons shared by both splice variants. Each intron contains “GU” dyad oligonucleotide at the 5' splice site and “AG” dyad oligonucleotide at the 3' splice site. In addition, the polyadenylation (polyA) signals (AAUAAA) downstream of each ORF are also found in the genomic sequence.

### Sequence analysis and phylogeny of *Spodoptera* Su(*var*) 3-9

**Figure 2:**
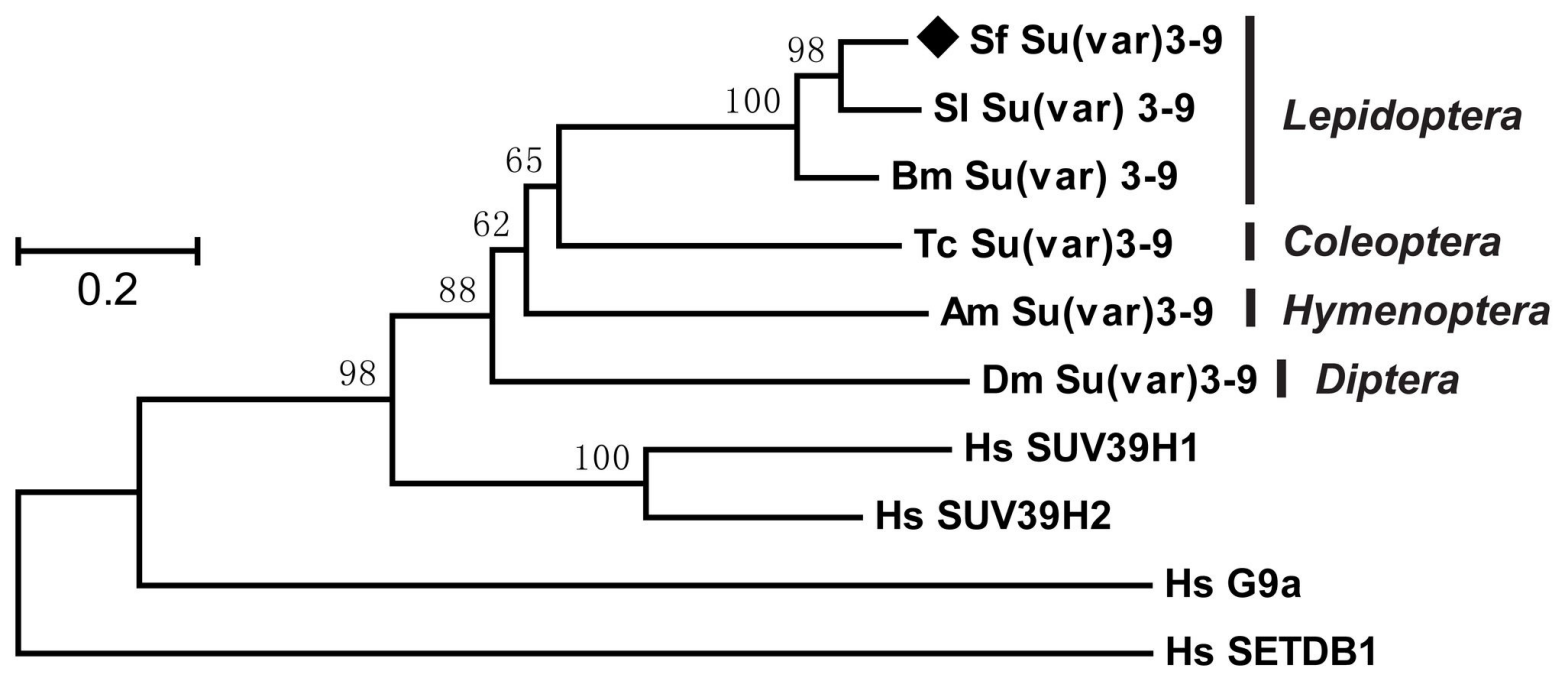
Phylogenetic tree of selected HMTs. Method: Neighbor-Joining, phylogeny test: Bootstrap (1 000 reps), substitution model: amino acid with Jones-Taylor-Thornton (JTT) model. Bootstrap values over 50% are indicated on the top of each node. The orders of insect species are indicated. *Spodoptera* Su(var) 3-9 is highlighted with prism. Abbreviations: Sf, *Spodoptera* *frugiperda* ; Sl*,* *Scoliopteryx* *libatrix* ; Bm, *Bombyx mori*; Tc, *Tribolium* *castaneum* ; Am, *Apis mellifera*; Dm, *Drosophila melanogaster*; Hs, *Homo sapiens*.

**Figure 3:**
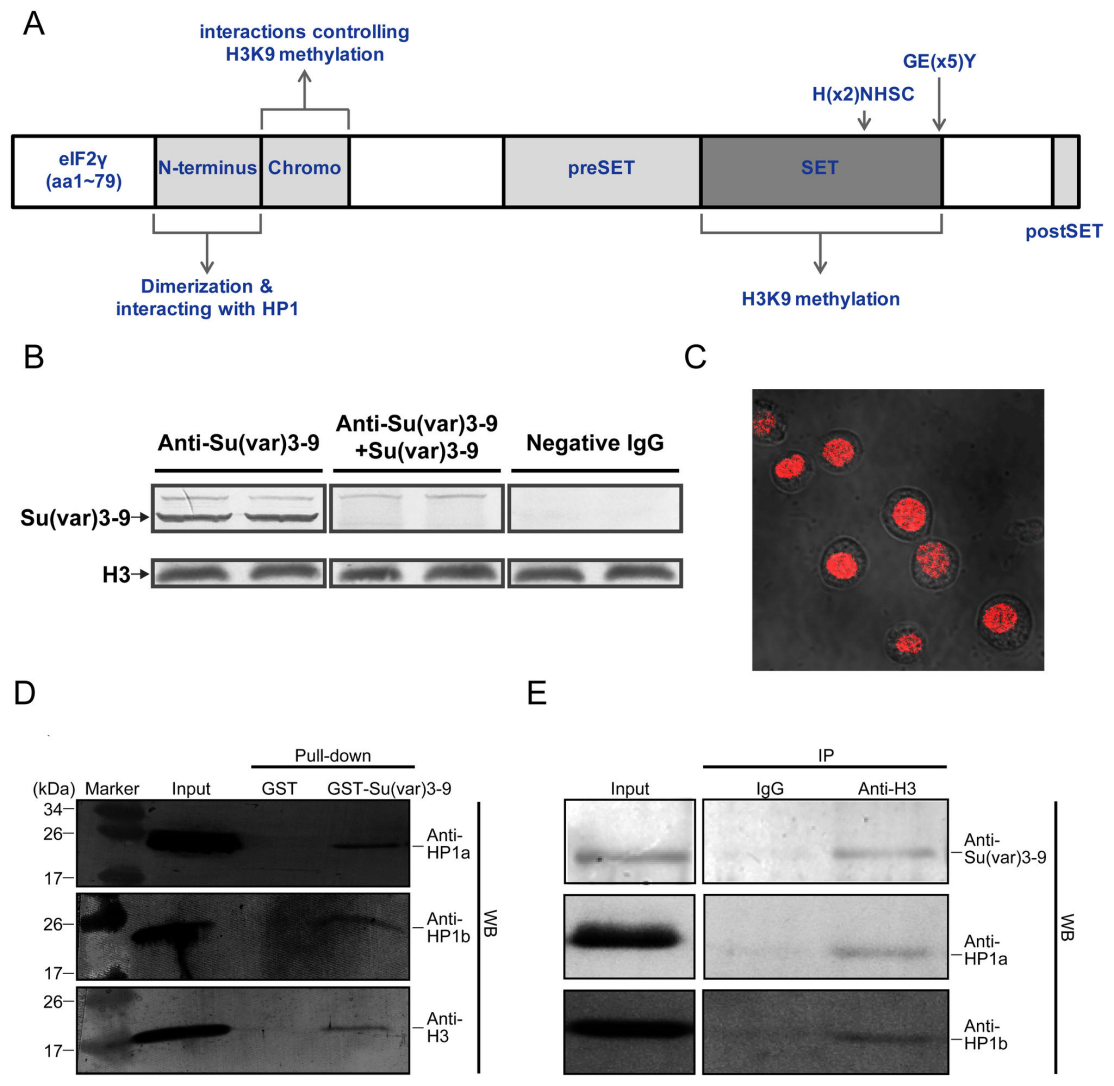
(A) Schematic diagram of *Spodoptera* Su(var) 3-9 secondary structure. (B, C) expression (B) and subcellular localization (C) of Su(var) 3-9 in Sf9 cells. (D, E) GST pull-down assay (D) and co-immunoprecipitation assay (E) to study the interactions between Su(var) 3-9, HP1a, HP1b and Histone H3. M: protein marker (prestained).

The amino acid sequence of *Spodoptera* Su(var) 3-9 was aligned with SUV39 family members from *Scoliopteryx* *libatrix* (Sl), *Bombyx mori* (Bm), *Tribolium* *castaneum* (Tc), *Apis mellifera* (Am), *Drosophila melanogaster* (Dm) and Homo sapiens (Hs). They cover insect species from the orders *Lepidoptera*, *Coleoptera*, *Hymenoptera* and *Diptera*, apart from human being. In the neighbor-joining phylogenetic tree, (Figure 2) SUV39 family members are well separated into each corresponding monophyletic group and the human SUV39s are distant from those of insect. The distantly related enzymes of SUV39 family, human G9a and SETDB1 are further apart from others, as expected. The three lepidopteran Su(var) 3-9s are most closely related (the identity between *Spodoptera* and *S* *. libatrix* Su(var) 3-9s is 74.20%, and the identity between *Spodoptera* and *B. mori* Su(var) 3-9s is 75.40%). According to the alignment of these three lepidopteran Su(var) 3-9s and the Su(var) 3-9 of *Drosophila* (Figure S1), the *Spodoptera* Su(var) 3-9 contains all conserved domains found in its insect counterparts (Figure 3A), including the common region with eIF2γ, the N-terminus of Su(var) 3-9, the Chromo domain, the preSET domain, the SET domain and the postSET domain [38,39]. Two highly conserved motifs, H(x_2_) NHSC and GE(x_5_) Y, both of which locate in the C-terminal half of the SET domain and are defined as catalytic core [4,5], are also present in *Spodoptera* Su(var) 3-9, as well as in other Su(var) 3-9s.

### Expression and localization of Su(*var*) 3-9 in Sf9 cells

Western blot was performed to detect *Spodoptera* Su(var) 3-9 in Sf9 cells using homemade antiserum generated with bacterially expressed Su(var) 3-9. A band with apparent molecular mass slightly higher than predicated (68 kDa) was seen (Figure 3B, left), most likely because of post-translational modifications such as phosphorylation [40]. No visible bands of Su(var) 3-9 were detected in the antigen-preabsorbed antiserum (obtained by mixing the anti-Su(var) 3-9 antiserum with excessive amount of purified Su(var) 3-9) and irrelevant mouse IgG groups, which verified the specificity of homemade antibody (Figure 3B, middle and right). Immunofluorescence analysis indicated that Su(var) 3-9 mainly localized in the nuclei in Sf9 cells (Figure 3C), which is consistent with its activity as HMT.

### Interactions of Su(*var*) 3-9 with histone H3 and heterochromatin proteins HP1a/b

In *Drosophila* , the histone H3K9me3 serves as a signal for the binding of HP1 [10–12]. Besides, the N-terminus of Su(var) 3-9 can also directly interact with the Chromo-shadow domain of HP1 [13,14]. GST pull-down assay was carried out to examine those interactions in *Spodoptera* using GST-tagged proteins and the nuclear extract of Sf9 cells. As expected, the interactions of Su(var) 3-9 with its substrate histone H3 and HP1a /b (see Figure S2 for detection and localization of HP1a/b in Sf9 cells) were seen, whereas no detectable bands were found in the negative control with the GST tag as the bait (Figure 3D). In addition, the specific interactions of Su(var) 3-9 and HP1a/b with histone H3 in Sf9 cells were also confirmed in the co-immunoprecipitation experiment (Figure 3E)

### Enzymatic activity assay in vitro and in vivo

**Figure 4:**
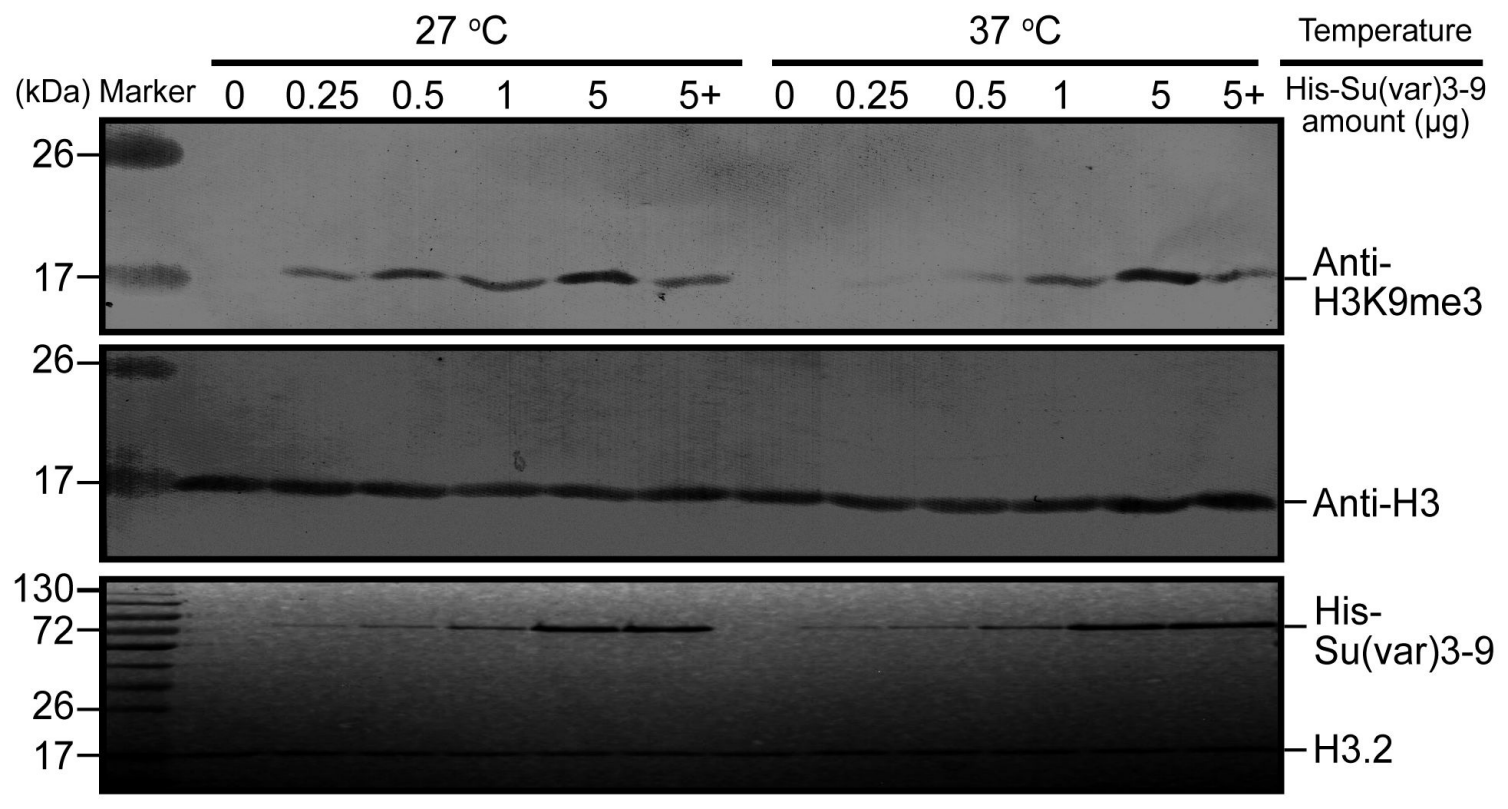
Enzymatic activity assay in vitro with serial amount of enzyme at 27 °C or 37 °C. 0.25-5 μg bacterially expressed *Spodoptera* His-Su(var) 3-9 (~70 kDa) was added in the reaction, the “5+” indicates the presence of 5 µM Chaetocin in the reactions containing 5 µg recombinant His-Su(var) 3-9. Reaction product was analyzed with Western blot and the bands of trimethylated H3K9 and total H3 (~18 kDa) were shown in the upper and middle panel, respectively. The SDS-PAGE of reaction mixtures was shown at bottom panel.

*In vitro* enzymatic activity assay was performed to determine the ability of *Spodoptera* Su(var) 3-9 for histone H3K9 trimethylation. Various amount of His-Su(var) 3-9 (0.25~5 µg) was added to the reaction systems containing same amounts of H3 and SAM. As shown in Figure 4, more trimethylated H3K9 were observed with the increased amount of His-Su(var) 3-9 at both 27 °C and 37 °C. Higher enzymatic activity was seen at 27°C than at 37 °C. In addition, when Su(var) 3-9 specific inhibitor chaetocin [34] was added, significant inhibition of the enzymatic activity was seen (Figure 4). In the presence of chaetocin, the level H3K9me3 of the 5µg of Su(var) 3-9 reaction group was equivalent to that of the 1µg group, at both temperatures tested.

**Figure 5:**
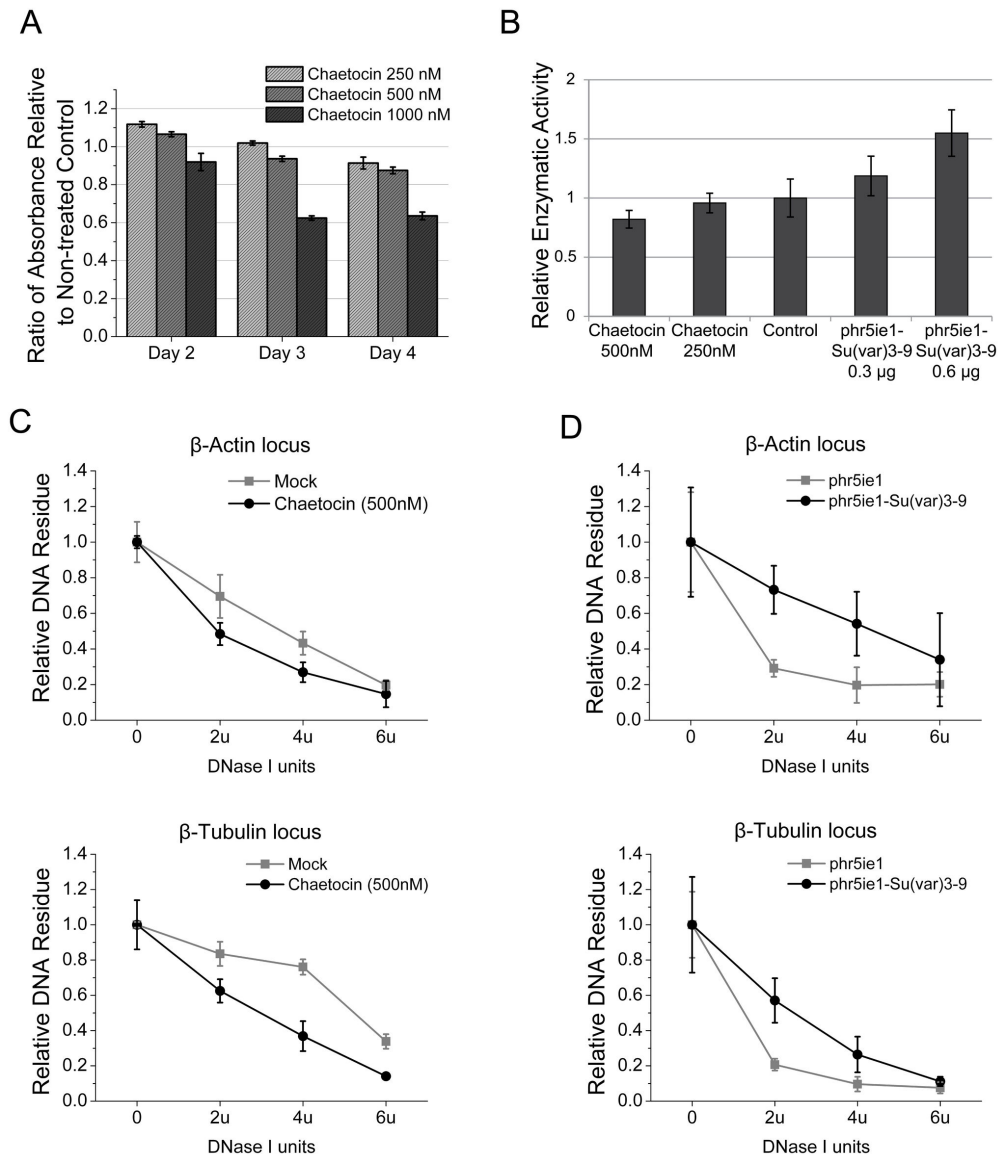
Activity of Su(var) 3-9 in Sf9 cells. Sf9 cells were harvested 3 d post treatments with Chaetocin at appropriate concentration determined by MTT assay (A) and transient overexpression of S. *frugiperda* Su(var) 3-9. Nuclear proteins were extracted and the global methylation level was evaluated (B). Cellular DNA was isolated and digested with DNase I. The percentage of DNA resistant to the nuclease was determined with qPCR with primers for S. *frugiperda β-Actin* and β*-Tubulin* (C, D). The error bars indicate the standard deviation calculated from at least three independent parallel experiments.

Cell viability assay showed that Sf9 cells could tolerate up to 500 nM chaetocin in the medium (Figure 5A). When the cells were treated with increased dosage of chaetocin, general histone methylation level was gradually inhibited, indicating the presence of Su(var) 3-9 activity in the cell (Figure 5B). In addition, the cellular DNA became more sensitive to DNase I at *β-Actin* and β*-Tubulin* loci, which reflected the decreased chromatin compaction (Figure 5C). In contrast, the opposite trends of results were observed when the cells were transfected with a plasmid overexpressing Su(var) 3-9 (Figure 5D).

### Effect of AcMNPV infection on Su(*var*) 3-9 expression

**Figure 6:**
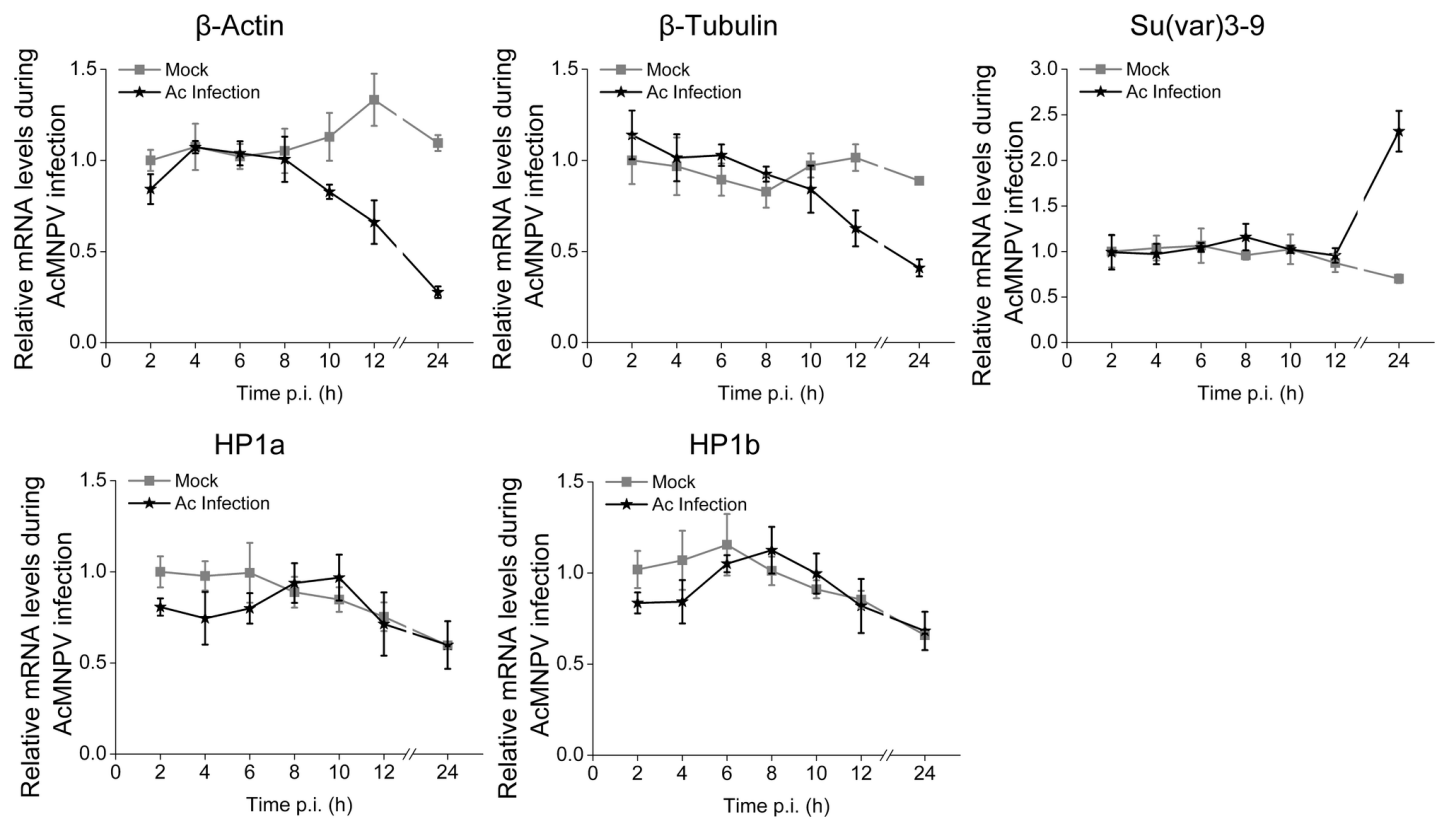
Influence of AcMNPV infection on the transcription of several host genes. Sf9 cells inoculated with AcMNPV at MOI=20 pfu/cell or mock infected were harvested at different time point post infection (p.i.). Total RNA was extracted, reverse transcribed and determined by qPCR. The transcription level of *β-Actin*, β*-Tubulin*, *Su*(*var*) *3-9*, *HP1a* and *HP1b* were normalized to *GAPDH*. The error bars indicate the standard deviation calculated from at least three independent infections.

**Figure 7:**
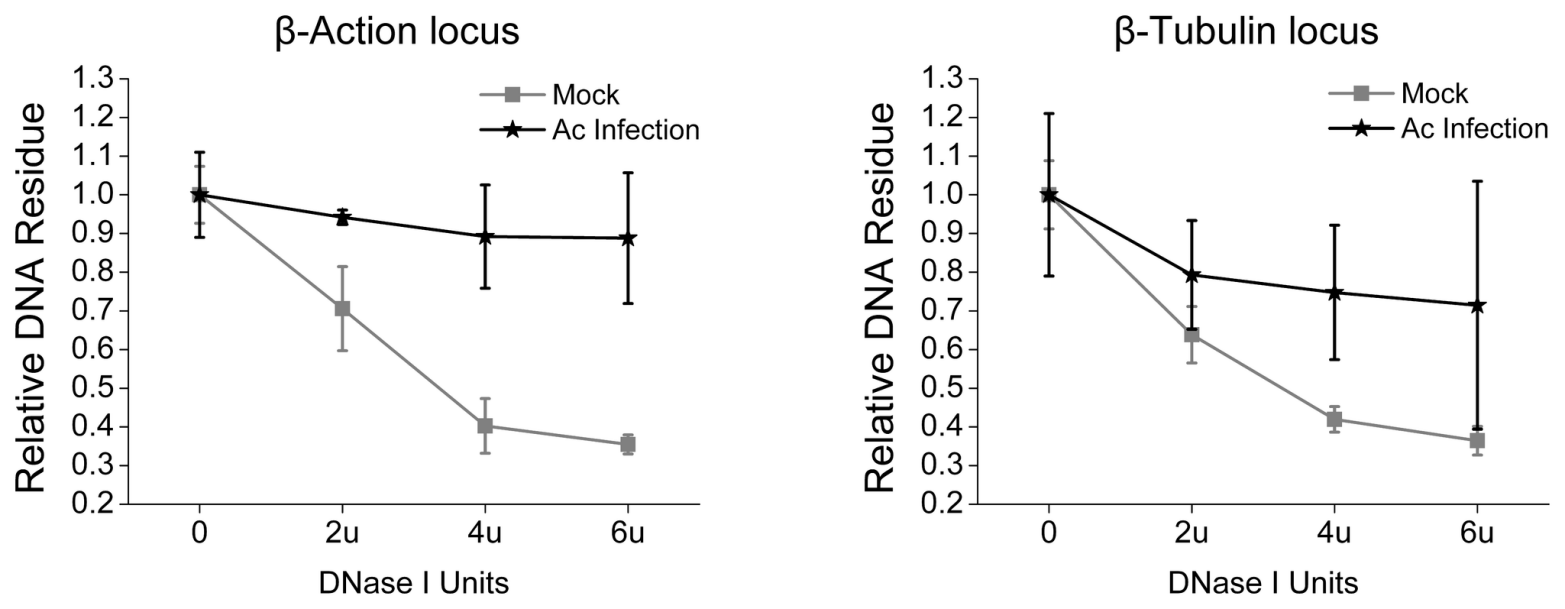
The effect of AcMNPV infection on host gene activity. Genomic DNA from AcMNPV or mock -infected cells were digested with DNase I. The percentage of DNA resistant to the nuclease was determined with qPCR with primers for S. *frugiperda β-Actin* and β*-Tubulin*. The error bars indicate the standard deviation calculated from at least three independent parallel experiments.

Whether Su(var) 3-9 is involved in AcMNPV infection and virus–host interaction is studied. AcMNPV-infected Sf9 cells (MOI=20 plaque-forming units per cell) were examined for Su(var) 3-9 transcription by qPCR. As shown in Figure 6, transcriptions of cellular cytoskeleton genes, *β-Actin* and β*-Tubulin* declined dramatically 8-12 h post infection (p.i.), whereas the mRNA level of *Su*(*var*) *3-9* increased to more than threefold higher than that of the control after 12 h p.i. The transcriptions level of *HP1a* and *HP1b* remain similar to those in the control. DNase I-sensitivity assay was executed to examine whether the abnormal expression trends of *Su*(*var*) *3-9*, *HP1a* and *HP1b*, in contrast with the majority of host genes that decreased their expression after virus infection, caused chromatin compaction. The results showed that the cellular DNA of infected Sf9 cells became less sensitive to DNase I than that of mock infected (Figure 7) at the loci of *β-Actin* and β*-Tubulin* genes 24 h p.i.

### Influence of Su(*var*) 3-9 on AcMNPV genome replication and genes transcription

**Figure 8:**
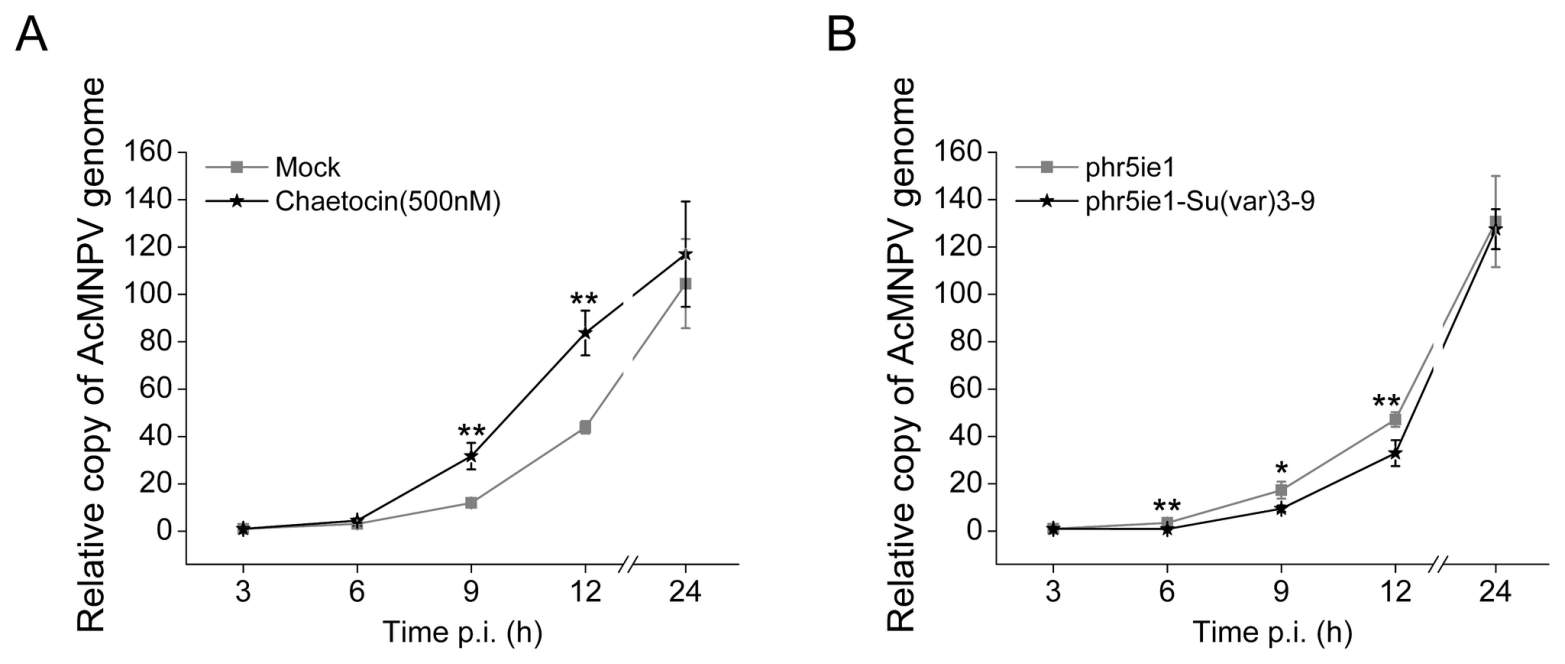
The effect of Chaetocin (A) and overexpression of Su(var) 3-9 (B) on AcMNPV DNA replication. The error bars indicate the standard deviation calculated from at least three independent parallel experiments. The asterisks indicate statistical significances between groups evaluated with student’s *t* test (∗P < 0.05; ∗∗P < 0.01).

**Figure 9:**
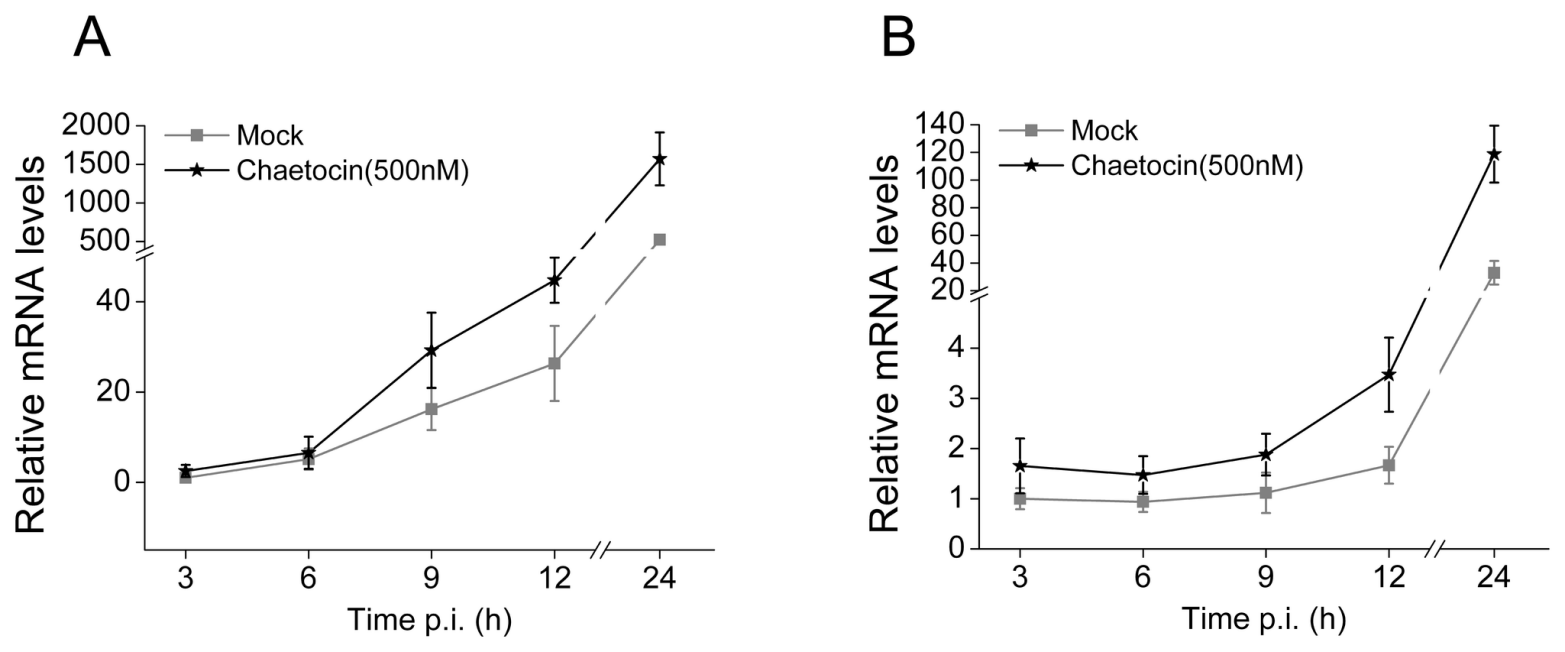
The effect of Chaetocin on the transcriptions of AcMNPV immediate early genes *IE0/1* (A) and *IE2* (B). The error bars indicate the standard deviation calculated from at least three independent parallel experiments. p.i.: post infection.

To evaluate the potential effect of Su(var) 3-9 on AcMNPV replication, Sf9 cells were treated with the chaetocin (500 nM) or transfected with Su(var) 3-9 overexpressing plasmid 24 h before being infected by AcMNPV (5 pfu/cell). Viral DNA replication and the transcription of representative genes, varying from immediate early to late stages, were examined with qPCR. As shown in Figure 8, AcMNPV genome replication was accelerated or delayed when treated with Chaetocin or transformed with overexpressing vector of Su(var) 3-9, respectively, although viral DNA replication achieved a similar level at the very late phase of infection in both cases. Minor increase in the expression of viral immediate early genes *IE0/1* (Figure 9A) and IE2 (Figure 9B) were seen. Variable increases in the transcription level were also observed for either early genes, including *lef1*, *lef2*, *lef3*, *dnapol*, *p35* and *gp64*, or the late genes *p78/83* (*orf1629*), *vp39* and *p10* (Table 1). The *lef2* gene was most dramatically activated with over 50-fold enhancement in transcription at 12 h p.i., whereas in the cases of *lef3* and *p35*, there were about 5 to 6-fold changes. The peak of transcription enhancement appeared before or at 12 h p.i. for most genes, except *P78/83* and *P10*, which appeared at 24h p.i.

**Table 1:** The effect of Chaetocin (500 nM) on the transcriptions of AcMNPV early genes *Lef1*, *Lef2*, *Lef3*, *DNA pol*, *P35*, and *Gp64*, late and very late genes *P78/83*, *Vp39* and *P10*.

Gene	Group		Time p.i. (h)	
		6	12	24
*Lef1*	Mock	1.00±0.08	0.83±0.20	12.21±3.61
	Chaetocin	14.43±5.34 (14)	29.00±14.74 (35)	246.55±36.87 (20)
*Lef2*	Mock	1.00±0.24	1.02±0.40	12.31±3.89
	Chaetocin	16.28±2.69 (16)	50.92±26.88 (50)	302.15±15.76 (25)
*Lef3*	Mock	1.00±0.25	0.73±0.28	9.99±3.46
	Chaetocin	7.21±0.74 (7)	3.86±1.91 (5)	19.96±3.48 (6)
*DNA Pol.*	Mock	1.00±0.22	0.61±0.10	7.26±0.75
	Chaetocin	10.35±1.56 (10)	14.78±9.33 (24)	147.85±51.00 (20)
*P35*	Mock	1.00±0.22	0.93±0.17	6.32±1.95
	Chaetocin	6.56±0.28 (7)	5.77±3.15 (6)	32.31±7.46 (5)
*Gp64*	Mock	/	1.00±0.16	4.44±2.16
	Chaetocin	/	9.51±3.33 (10)	37.54±6.94 (8)
*P78/83*	Mock	/	1.00±0.40	11.99±4.34
	Chaetocin	/	26.69±18.92 (27)	427.16±171.81 (35)
*Vp39*	Mock	/	1.00±0.22	4.63±2.50
	Chaetocin	/	23.41±7.77 (23)	94.08±15.02 (20)
*P10*	Mock	/	1.00±0.23	5.13±1.99
	Chaetocin	/	19.52±9.19 (20)	158.29±23.29 (31)

Data is shown as mean value plus/minus standard deviation calculated from at least three independent parallel experiments. Numbers in brackets represent mean value ratio of treated to non-treated (mock) group at each time post infection (p.i.).

## Discussion

In the current study, the histone H3K9 trimethyltransferase gene *Su*(*var*) *3-9* was identified in three *Spodoptera* insects, *S* *. frugiperda* , *S.* exigua and *S* *. litura* . As in other holometabolic insects, *Su*(*var*) *3-9* overlaps with *eIF2γ* within the same genome loci, and the two genes are expressed by alternative splicing [38,39]. Phylogeny analysis revealed that *Spodoptera* Su(var) 3-9 is highly related to selected SUV39 family members of insects from *Lepidoptera*, *Coleoptera*, *Hymenoptera*, and *Diptera*. It is also homologous to human SUV39. It sits closely with the two other *Lepidoptera* Su(var) 3-9s from *Scoliopteryx* *libatrix* (also belonging to the family of *Noctuidae* as *Spodoptera* ) and *Bombyx mori* (belonging to the family of *Bombycidae*), whereas the two distantly related SUV39 family members, human G9a and SETDB1, locate farthest from others.

Among numerous HMTs for histone lysine or arginine modifications, Su(var) 3-9 has the specific catalytic capability to catalyze histone H3K9me3. This activity was confirmed for *Spodoptera* Su(var) 3-9 *in vitro* and in Sf9 cells. *Spodoptera* Su(var) 3-9 exhibits a dose-dependent enzymatic activity at both 27 °C and 37 °C, but higher activity was seen at 27 °C in the lower enzyme concentration groups *in vitro*, coinciding with the fact that 27 °C is close to the optimal temperature for the insects.

*Spodoptera* Su(var) 3-9 was also proved to interact with histone H3 and HP1a/b, and the presence of Su(var) 3-9 specific inhibitor Chaetocin or Su(var) 3-9 overexpressing vector change the chromatin compaction in Sf9 cells accordingly. This was consistent with previous studies in other species that histone H3K9me3 catalyzed by Su(var) 3-9 may serve as a signal for HP1 binding and HP1 can subsequently recruit more Su(var) 3-9 to modify neighboring histones, thus causing the formation and diffusion of heterochromatin [13].

It has been reported that AcMNPV infection may provoke multiple host responses including general down regulation of cellular mRNA level and shut-down of host protein synthesis [24,25]. This was supported by the results of DNase I sensitivity assay, which showed that host chromatin became more compacted after AcMNPV infection. Surprisingly, the transcription of *Su*(*var*) *3-9* increased in AcMNPV-infected Sf9 cells at late time of infection, while the transcriptions of functionally related *HP1a* and *HP1b* were comparable to mock infected Sf9 cells. The detailed mechanism needs further clarification.

The genomes of a variety of DNA viruses are subjected to nucleosome assembly and are associated with histone modifications, which may inevitably take effect on viral life cycle. Even the acutely replicating DNA viruses such as Adenoviruses, which encode their own DNA polymerase and viral DNA assembly proteins, do not escape chromatin assembly. This might be helpful for viruses for their proper temporal control of viral gene transcription and genome stability during nuclear entry and the early stages of virus replication. Taken together with the previous reports that AcMNPV genome also has nucleosome-like structure during infection, and that several epigenetic drugs such as TSA, NaBu and DAC have significant effect on viral genome replication and gene transcription [26–29], it is reasonable to believe that changes in histone modification may have a role in AcMNPV genome replication and gene transcription. In this study we demonstrated that Su(var) 3-9 specific inhibitor Chaetocin or transient expression of Su(var) 3-9 could activate or repress AcMNPV genome replication and gene transcription, respectively, suggesting that Su(var) 3-9 is possibly involved in AcMNPV infection. It deserves more detailed study due to the complex epigenetic regulation network.

## Supporting Information

Figure S1 Alignment of amino acid sequence of Su(var) 3-9s from *S* *. frugiperda* (Sf), *Scoliopteryx* *libatrix* (Sl), *Bombyx mori* (Bm), and *Drosophila melanogaster* (Dm). Identical residues are dark black shadowed with white font. Similar residues are gray shadowed. The regional subdivisions of common region with eIF2γ, N-terminus of Su(var) 3-9 Chromo domain, preSET, SET and postSET are indicated within black framed below the sequence. The catalytic core motifs of H(x_2_) NHSC and GE(x_5_) Y are marked with red asterisks.
(TIF)

Figure S2 Expression and subcellular localization of HP1a/b in Sf9 cells. Representative individuals are marked with light green arrows in the immunofluorescence pictures. M: protein marker (prestained).
(TIF)

Table S1 Information of genes used in this research. (DOCX)

Table S2 Primers used in fragments amplification for plasmids construction. (DOCX)

Table S3 Primers used in sequences determination. (DOCX)

Table S4 Primers used for Real-time quantitative PCR. (DOCX)
