# Supplementary figures and images for: Identification and Characterization of the Spodoptera Su(var) 3-9 Histone H3K9 trimethyltransferase and Its Effect in AcMNPV Infection
Source: PLoS One. 2013 Jul 24;8(7):e69442. doi: 10.1371/journal.pone.0069442 (PMC3722159; doi:10.1371/journal.pone.0069442)

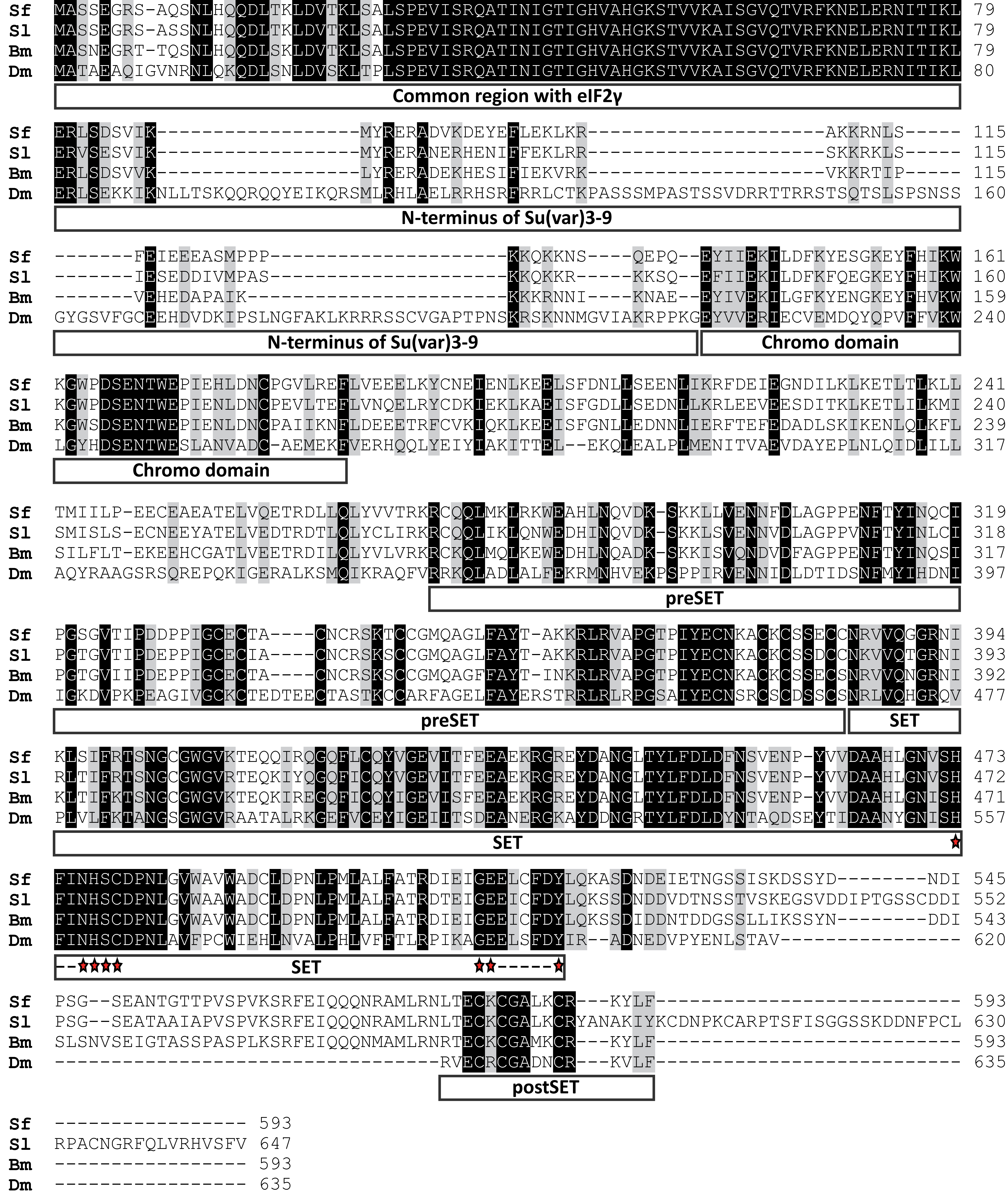

Supplement: Figure S1 — Identical residues are dark black shadowed with white font. Similar residues are gray shadowed. The regional subdivisions of common region with eIF2γ, N-terminus of Su(var) 3-9 Chromo domain, preSET, SET and postSET are indicated within black framed below the sequence. The catalytic core motifs of H(x2) NHSC and GE(x5) Y are marked with red asterisks. (TIF) [file pone.0069442.s001.tif]

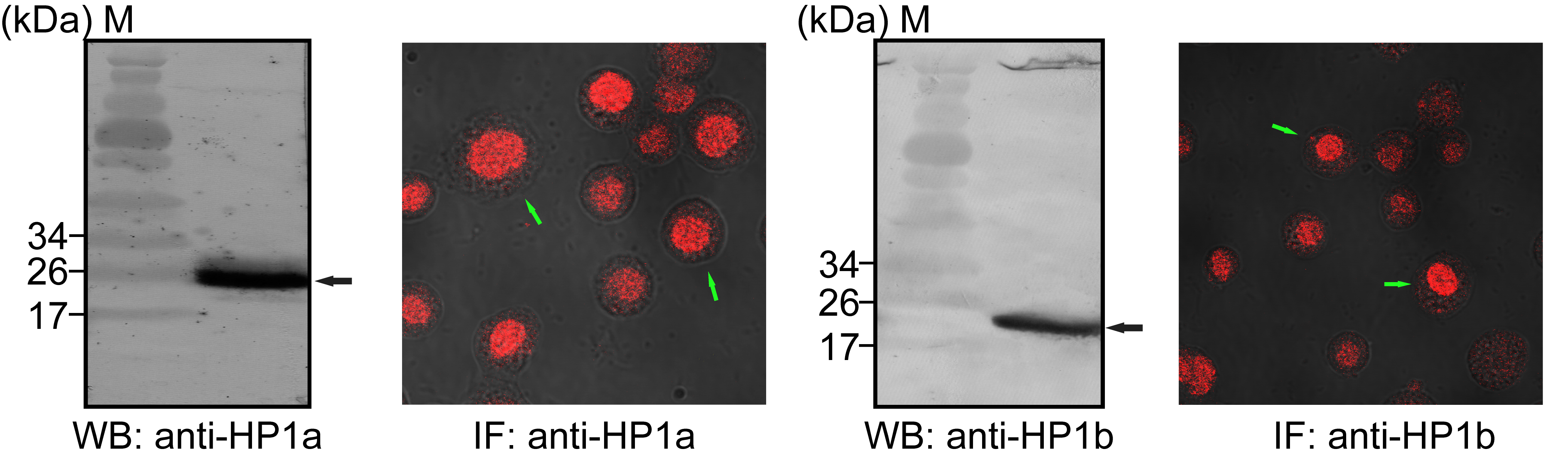

Supplement: Figure S2 — Representative individuals are marked with light green arrows in the immunofluorescence pictures. M: protein marker (prestained). (TIF) [file pone.0069442.s002.tif]
