# Supplementary material for: Identification and Characterization of the Spodoptera Su(var) 3-9 Histone H3K9 trimethyltransferase and Its Effect in AcMNPV Infection
Source: PLoS One. 2013 Jul 24;8(7):e69442. doi: 10.1371/journal.pone.0069442 (PMC3722159; doi:10.1371/journal.pone.0069442)
Supplement: Table S1 — (DOCX) [file pone.0069442.s003.docx]

| ***Species*** | ***Abbrev.*** | ***Gene*** | ***Database*** | ***Accession number*** |
| --- | --- | --- | --- | --- |
| *Bombyx mori* | Bm | *Su(var)3-9* | NCBI | NP_001037070.1 |
|  |  | *HP1a* | NCBI | NP_001040539.1 |
|  |  | *HP1b* | NCBI | NP_001159616.1 |
| *Spodoptera frugiperda* | Sf | *Su(var)3-9/eIF2γ* | SPODOBASE | Sf2H06915-5-1 |
|  |  | *HP1a* | SPODOBASE | Sf1P08238-5-1 |
|  |  | *HP1b* | SPODOBASE | Sf2M03114-5-1 |
| *Scoliopteryx libatrix* | Sl | *Su(var)3-9* | NCBI | GI_9409736 |
| *Tribolium castaneum* | Tc | *Su(var)3-9* | NCBI | GI_270002141 |
| *Apis mellifera* | Am | *Su(var)3-9* | NCBI | GI_94420672 |
| *Drosophila melanogaster* | Dm | *Su(var)3-9* | NCBI | GI_24647050 |
| *Homo sapiens* | Hs | *SUV39H1* | NCBI | GI_4507321 |
|  |  | *SUV39H2* | NCBI | GI_301171588 |
|  |  | *G9a* | NCBI | GI_287865 |
|  |  | *SETDB1* | NCBI | GI_224177467 |

Abbrev.: Abbreviation
