# Supplementary material for: Identification and Characterization of the Spodoptera Su(var) 3-9 Histone H3K9 trimethyltransferase and Its Effect in AcMNPV Infection
Source: PLoS One. 2013 Jul 24;8(7):e69442. doi: 10.1371/journal.pone.0069442 (PMC3722159; doi:10.1371/journal.pone.0069442)
Supplement: Table S2 — (DOCX) [file pone.0069442.s004.docx]

| ***Primers from SMARTer RACE cDNA Amplification Kit (5`→3`)*** | |
| --- | --- |
| ***Primers for amplification of Su(var)3-9 ORF excluding ATG (5`→3`)*** | |
| CommonF(-ATG) | GCGGGATCCGCTTCGAGTGAAGGGCGAAGTGCTC |
| Su(var)3-9R | GCGCTCGAGTCATTAAAATAAATATTTGCGACAT |
| ***Primers for amplification of HP1a ORF excluding ATG (5`→3`)*** | |
| HP1aF(-ATG) | GCGGGATCCGGTAAAAAGGATAAAAAAGGTG |
| HP1aR | GCGCTCGAGTTCTGACTCCGCCGGTGT |
| ***Primers for amplification of HP1b ORF excluding ATG (5`→3`)*** | |
| HP1bF(-ATG) | GCGGGATCCGCTGACAAGAAAAAGGAAGG |
| HP1bR | GCGCTCGAGGTCATCGGCGCCAGCTTCAT |
| ***Primers for amplification of Su(var)3-9 ORF excluding common region with eIF2γ(5`→3`)*** | |
| SuvF | GCGGGATCCCGCCTGTCGGACTCAGTTA |
| Su(var)3-9R | SEE ABOVE |
| ***Primers for amplification of eIF2γ ORF excluding common region with Su(var)3-9 (5`→3`)*** | |
| eIFF | GCGGGATCCTGTGACAATCCGAAATGTC |
| eIF2γR | GCGCTCGAGTCATTAATTCTTTGCTGGCTCGATTG |
| ***Primers for amplification of hr5ie1 fragment*** | |
| hr5ie1F | GGCGTATACCGGCGCGTAAAACACAATCA |
| hr5ie1R | GCGACTAGTTCTTGTCGCCGCCAGTGTCA |
