# Supplementary material for: Identification and Characterization of the Spodoptera Su(var) 3-9 Histone H3K9 trimethyltransferase and Its Effect in AcMNPV Infection
Source: PLoS One. 2013 Jul 24;8(7):e69442. doi: 10.1371/journal.pone.0069442 (PMC3722159; doi:10.1371/journal.pone.0069442)
Supplement: Table S3 — (DOCX) [file pone.0069442.s005.docx]

| ***Primers used for 5`-RACE of Su(var)3-9/eIF2γ(5`→3`)*** | |
| --- | --- |
| GSPs/e51 | GGGCACGCTATCTCCGTTCATTTCTAT |
| GSPs/e52 | ATGTAGCTCATGGTAAATCAACCGTAGT |
| ***Primers used for 3`-RACE of Su(var)3-9/eIF2γ(5`→3`)*** | |
| GSPs/e3 | CCCTGGGCACGACATTCTTATGGCAACC |
| ***Primer used for 5`-RACE of HP1a (5`→3`)*** | |
| GSPa5 | AGCTACTCTAGCAGGCACCAG |
| ***Primer used for 3`-RACE of HP1a (5`→3`)*** | |
| GSPa3 | GAACGCAAGGAAGACAAATCA |
| ***Primer used for 5`-RACE of HP1b (5`→3`)*** | |
| GSPb5 | GTTGGCACAGCAGATTTACGC |
| ***Primer used for 3`-RACE of HP1b (5`→3`)*** | |
| GSPb3 | AAAGGTTACAATGACGAGGACA |
| ***Primers for mapping Su(var)3-9/eIF2γ in spodoptera exigua and spodoptera litura (5`→3`)*** | |
| s/eF | GCTTCGAGTGAAGGGCGAAGTGCTC |
| sR | TTAAAATAAATATTTGCGACAT |
| eR | CATTAATTCTTTGCTGGCTCGATTG |
| ***Primers used for mapping Su(var)3-9/eIF2γ locus within genome context (5`→3`)*** | |
| GeneF1 | GGGCACGCTATCTCCGTTCATTTCTAT |
| GeneR1 | TGGCCTTCCTTGACCAAATCTATCTTA |
| GeneF2 | GTGCTCCTCAGAGTGCTGTAAT |
| GeneR2 | TTAAAATAAATATTTGCGACAT |
