# Supplementary material for: Identification and Characterization of the Spodoptera Su(var) 3-9 Histone H3K9 trimethyltransferase and Its Effect in AcMNPV Infection
Source: PLoS One. 2013 Jul 24;8(7):e69442. doi: 10.1371/journal.pone.0069442 (PMC3722159; doi:10.1371/journal.pone.0069442)
Supplement: Table S4 — (DOCX) [file pone.0069442.s006.docx]

| ***Target*** | ***Sequence (5`→3`)*** | ***Source*** |
| --- | --- | --- |
| *Su(var)3-9* | Forward: CGCCTGTCGGACTCAGTTAT  Reverse: GAGGCTCCTGGGAATTTTTC | *S. frugiperda* |
| *HP1a* | Forward: GAGAGCACATGGGAACCAGT  Reverse: TCCCTCTTGGAGTCCTTGTG | *S. frugiperda* |
| *HP1b* | Forward: GGCTACAATGACGAGGAC  Reverse: GGAGTTGGCACAGCAGAT | *S. frugiperda* |
| *GAPDH* | Forward: CGGTGTCTTCACAACCACAG  Reverse: GAGAGGAGCGAGACAGTTGG | *S. frugiperda* |
| *β-Actin* | Forward: TCAACCCCAAGGCCAACAGAGA  Reverse: GACCGGACGCGTACAGGGACAG | *S. frugiperda* |
| *β-Tubulin* | Forward: TTGCATTGGTACACTGGCGA  Reverse: ACACCAGGTCGTTCATGTTGC | *S. frugiperda* |
| *IE0/1* | Forward: TTTTAACGCGTCGTACACCA  Reverse: GTTGACGCTTGCCAAAAAGT | AcMNPV |
| *IE2* | Forward: AGCGAAGAAAACGTGCAGAT  Reverse: CTCCGACGCAATGTTATCCT | AcMNPV |
| *Lef1* | Forward: TTCCGCATATGCAAGATTCA  Reverse: ACCACATCCACCAGTTCCAT | AcMNPV |
| *Lef2* | Forward: ATTTCCGGACTGCGATTGTA  Reverse: GCGCGGTACATTGTTTTCTT | AcMNPV |
| *Lef3* | Forward: CCGATTCGGATGACTGTTCT  Reverse: GTTGTGATTCTCGCCGTTCT | AcMNPV |
| *DNA pol.*  *(DNA polymerase)* | Forward: AAACACGCGCATTAACGAGAGCAC  Reverse: ATTGGCCCGATATTATTGACAGAT | AcMNPV |
| *P35* | Forward: ACGACACGGGACTTTACGAG  Reverse: GTTTTTCGACGCTTCGTTGT | AcMNPV |
| *Gp64* | Forward: CTGCAAAAGGACGTGGAAAT  Reverse: GAAAACAGTCGTCGCTGTCA | AcMNPV |
| *P78/83*  *(ORF 1629)* | Forward: AATATCGGCACGGTAAACGA  Reverse: GAATGTTAGGCACGGGAGAA | AcMNPV |
| *Vp39* | Forward: TTCGACGCGTGCATAACATACA  Reverse: TGCCTAGCGATCGTCATTTTG | AcMNPV |
| *P10* | Forward: GACGCCGTTACGGAAACTAA  Reverse: GTCTGGAAGATCCGGAACAA | AcMNPV |
